# Real-Time Relative qPCR without Reference to Control Samples and Estimation of Run-Specific PCR Parameters from Run-Internal Mini-Standard Curves

**DOI:** 10.1371/journal.pone.0011723

**Published:** 2010-07-22

**Authors:** Jens Magnus Bernth Jensen, Mikkel Steen Petersen, Marc Stegger, Lars J. Østergaard, Bjarne K. Møller

**Affiliations:** 1 Department of Clinical Immunology, University Hospital of Aarhus, Skejby, Denmark; 2 Department of Infectious Diseases, University Hospital of Aarhus, Skejby, Denmark; Natural History Museum of Denmark, Denmark

## Abstract

**Background:**

Real-Time quantitative PCR is an important tool in research and clinical settings. Here, we describe two new approaches that broaden the scope of real-time quantitative PCR; namely, run-internal mini standard curves (RIMS) and direct real-time relative quantitative PCR (drqPCR). RIMS are an efficient alternative to traditional standard curves and provide both run-specific and target-specific estimates of PCR parameters. The drqPCR enables direct estimation of target ratios without reference to conventional control samples.

**Methodology/Principal Findings:**

In this study, we compared RIMS-based drqPCR with classical quantifications based on external standard curves and the “comparative Ct method”. Specifically, we used a raw real-time PCR dataset as the basis for more than two-and-a-half million simulated quantifications with various user-defined conditions. Compared with classical approaches, we found that RIMS-based drqPCR provided superior precision and comparable accuracy.

**Conclusions/Significance:**

The obviation of referencing to control samples is attractive whenever unpaired samples are quantified. This may be in clinical and research settings; for instance, studies on chimerism, TREC quantifications, copy number variations etc. Also, lab-to-lab comparability can be greatly simplified.

## Introduction

Real-time relative quantitative polymerase chain reaction (qPCR) has long been a favoured principle for relative quantifications of nucleic acid sequences. In essence, an undetectably low amount of a specific nucleic acid target sequence is expanded by PCR to a measurable level. Subsequently, the original amount of the target sequence is calculated from the parameters of the PCR. The basis of these calculations is the classical PCR equation:
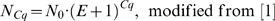
(I.1)
*N*_0_ is the amount of the target sequence before PCR, *N_Cq_* is the amount of target after *C_q_*-rounds of PCR, and *E* is the efficiency of the PCR-amplification. Usually, *E* is assumed constant until the onset of PCR exhaustion. The designation “relative quantification” refers to the fact that the amount of the target sequence is estimated relative to that of another (or several [2]). Applying the above relationship, the ratio before PCR of sequences *A* and *B* in a given interest-sample (*is*) can thus be estimated as:
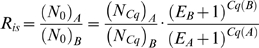
(I.2)To solve the equation above, *N_Cq_*s, *C_q_*s and *E*s must be accounted for. Indirect measures of *N_Cq_*s are estimated by fluorescence sampling. Various technologies exist (reviewed in [3], [4]), but the common principle of Real-Time PCR is to obtain fluorescence emissions of an intensity proportional to the amount of target at a given point of time [5]. *N_Cq_* and the corresponding *C_q_* are indirectly defined in the setting of a fluorescence-intensity threshold value. The threshold can be set by various approaches; for example, second-derivatives-maximum method, manually setting, and so on. In mainstream qPCR, *E* is either assumed to have a value of 1 (the “comparative Ct method” or “2^ΔΔ*Cq*^” [6], [7] or estimated target-specifically from a standard curve (SC) [8].

Despite the broad applicability of the technology, several methodological limitations have yet to be addressed. In this paper, we focus on two of these.

A general limitation is that **Eq.I.2** cannot be solved trivially offhand. The problem lies in the *N_Cq,A_*/*N_Cq,B_*-term; in conventional settings, the value of this term is unknown. This may not be problematic as such, provided that the ratio of *A* and *B* in the interest-sample is of interest *only* relative to their ratio in a control-sample (*cs*):
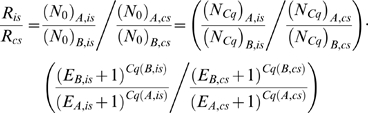
(I.3)The “double-ratio” above can be simplified, eliminating the *N_Cq,A_*/*N_Cq,B_*-term, if both *N_Cq_* and *E* are preserved for each target between the samples *is* and *cs*; that is: (*N_Cq_*)*_A,is_*/(*N_Cq_*)*_B,is_* = (*N_Cq_*)*_A,cs_*/(*N_Cq_*)*_B,cs_* and *E_A,is_* = *E_A,cs_*, *E_B,is_* = *E_B,cs_*):
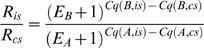
(I.4)Although appropriate for paired samples, **Eq. I.4** is generally unsuitable if samples are unpaired: the ratio of *A* and *B* in a sample can be of immediate interest, and any reference to a control sample can be inconvenient or even meaningless. In these situations, **Eq. I.4** can still be useful if the control sample contains *A* and *B* in equal numbers. Nonetheless, two problems remain. First, such control samples are not necessarily available. Second, and more importantly, the sources of errors increases by the doubling of sample numbers to be determined with **Eq. I.4** compared with **Eq. I.2**. Ultimately, increased error of the final ratio-estimate is very likely. It may therefore be attractive to actually use **Eq. I.2** directly. If so, the inherent (*N_Cq_*)*_A_*/(*N_Cq_*)*_B_*-term must be accounted for. Of course, if (*N_Cq_*)*_A_* and (*N_Cq_*)*_B_* happen to be equal, the term disappears, but this situation is unlikely to occur by chance. Simply assuming equality (i.e., ignoring the term entirely) induces proportional errors of the *R_is_*-estimate. “Ensuring” equality by assigning the same fluorescence-threshold-value for *A* and *B* is unreliable. The proportionality factor between fluorescence and sequence-numbers (*k*) differs widely between targets. For example, the *k*-value of the fluorophore SYBRGreen depends on the length and sequence of the amplicon as well as other factors, such as salts and temperature [9]. Use of sequence-specific probes is most likely subject to similar shortcomings, in addition to potential target-associated differences in probe-labelling efficiencies and fluorophore bleaching. The most reliable approach is therefore to obtain actual (*N_Cq_*)*_A_*/(*N_Cq_*)*_B_*-estimates, yet conventional approaches fall short in this respect.

Another important limitation of conventional approaches concerns the use of low-capacity machinery. Conventionally, limited instrument capacity forces the investigator to estimate PCR unknowns (such as *E*) from standard curves analysed in separate runs or assuming a value of 1. This introduces a run-to-run variability that inevitably contributes to the error of *E*. As such, *E* varies considerably between replicate runs (e.g. >5% [10]). Even tiny errors of the *E*-estimate are critical. These errors induce disproportionately large errors of *R_is_*/*R_cs_* or *R_is_* because *E* constitutes the base of the exponential PCR function (**Eq. I.1**). In this light, errors associated with run-to-run variability of PCR unknowns are highly undesirable.

We hypothesized that run-internal estimation of PCR unknowns (from small amounts of standard curve samples) is superior to run-external estimation. Also, by modifying the composition of standard curve samples, we hypothesized that target ratio estimates can be attained directly without reference to control samples.

Our objective was to deduce the optimal composition of standard curve samples to remedy the limitations of classical qPCR. In the process, we wanted to compare the precision and accuracy of our approach to classical approaches.

## Materials and Methods

### Construction of a fusion-PCR product

Blunt-ended PCR products of parts of Human Endogenous retrovirus 1 (ERV1) and TUP-like enhancer of SPLIT 1 (TUPLE1) were produced from genomic DNA by conventional PCRs (Platinum Pfx DNA Polymerase (InVitrogen)). Primer sequences were obtained from Overhauser J et al [11] and Weksberg R et al [12]. PCR products were gel electrophorized. Single bands of expected lengths were excised and PCR products purified (Illustra GFX™ PCR DNA and Gel Band Purification Kit, GE Healthcare). TUPLE1 PCR products were 5′-dephosphorylated with rAPID Alkaline Phospatase in supplied buffer (Roche) and purified. Blunt-ended ERV1 PCR products- and 5′-dephosphorylated TUPLE1 PCR products were ligated (T4 DNA Ligase in T4 DNA Ligase Buffer, New England Biolabs) and purified. Hundred-fold diluted fused PCR products were expanded by standard PCR (ERV1 forward and TUPLE1 reverse primers, respectively). Reactions were gel electrophorized; and single bands consistent with the expected length of the fused PCR product were obtained, purified, and diluted 100-fold before an additional round of PCR, isolation, purification, and dilution. The PCR products were validated by sequencing.

### Dilution series

The diluted fusion-PCR product was thoroughly mixed and stored in aliquots (−20°C). On three separate days, an aliquot was thawed and used for a 10-fold dilution series in eight steps. Weight data of the pipetted volumes were sampled while the dilution series were made. Each dilution step was performed three times into the same tube (to minimize impact of stochastic errors).

### Real-Time PCR

Each dilution series was analyzed by real-time PCR with the primer pairs for ERV1 and TUPLE1. Each primer pair was used in separate runs. Reactions of 20 µl were set up in LightCycler capillaries: 10 µl 2× QuantiTect SYBR Green PCR mix (Qiagen), 0.5 µM primers and 8 µl template. PCRs were conducted on a LightCycler 1.0 Instrument (Roche) with the following settings: 15 min at 95°C, 45 amplification cycles (each 15 seconds at 94°C, 20 seconds annealing at 57°C, and 20 seconds at 72°C with endpoint fluorescence detection). Each of the eight concentrations of the dilution series was analysed in four replicates. Six preliminary data sets containing 32 data points each were thus generated.

### Raw data sampling

We estimated *C_q_*s for the six preliminary data sets by the fit-points-approach, which gave more linear standard curves than the second derivative maximums-method (see **Appendix S1**, section 3). In practice, arithmetic baseline adjustment was used, noise bands were set by default, and the lowest (eighth) concentration that yielded *C_q_*s for all replicates was excluded (explained below). The remaining data points of the six separate data sets (seven concentrations, each analysed in replicates of four) were used in the “minimize error”-function of the LightCycler software (version 3.5) for threshold setting. The fit-point number was selected for each of the six data sets as the number providing the smallest error. Relative target concentrations (*N*_0_s) in the dilution-series-samples were determined from the loaded volumes unless otherwise stated.

### Data handling

Data handling was done in Microsoft Excel 2007. Sampled *C_q_*- and *N*_0_-data were used to generate standard curves. We excluded data obtained from the eighth dilution step to avoid introduction of heteroscedasticity, which would invalidate conventional linear regression analysis (see **Appendix S1**, section 1 and **Figure S1**). The data of the eighth concentrations thus merely represented a safety feature and were not used for further analysis. Standard curves based on relative target concentrations were defined as:
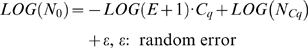
(M.1)Regarding LOG (*N*_0_) as the outcome variable and *C_q_* as the predictor variable is opposite of the conventional approach and may seem awkward. However, as *N*_0_ of the-samples-to-be-quantified are to be estimated as regression estimates from *C_q_* the perception makes sense. More importantly, estimating errors of *R_is_* is simpler as detailed in later sections and **Appendix S1** section 2. Thus, the formula for the regression equation is:
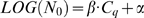
(M.2)With *α* and *β* being intercept and slope, respectively. Data of the six 28-sample- standard curves generated from fusion-PCR products are presented in **Table 1**.

**Table 1:** Data of the six 28-sample relative standard curves used in the study.

Target	Day	σ^2^	*β*	*α*	*E*	*N_Cq_*	*F_Cq_*	*k*	*K_pr BP_*
**ERV1**	**1**	**0.0070**	**-0.292**	**1.24**	**0.959**[0.938;0.981]	**17**[15;21]	**0.991**	**17.5**	**0.103**
	**2**	**0.0114**	**-0.285**	**2.11**	**0.928**[0.902;0.954]	**129**[99;167]	**5.89**	**21.8**	**0.128**
	**3**	**0.0057**	**-0.280**	**2.14**	**0.905**[0.887;0.923]	**139**[115;167]	**6.35**	**21.9**	**0.129**
**TUPLE1**	**1**	**0.0016**	**-0.294**	**1.28**	**0.967**[0.956;0.977]	**19**[17;21]	**2.89**	**6.59**	**0.066**
	**2**	**0.0025**	**-0.296**	**2.52**	**0.976**[0.963;0.989]	**334**[293;381]	**30.0**	**11.1**	**0.111**
	**3**	**0.0011**	**-0.286**	**2.36**	**0.931**[0.923;0.939]	**231**[212;252]	**27.6**	**8.37**	**0.084**

*N_Cq_* and *E* are presented with 95% confidence intervals. *N_Cq_*s are not in absolute numbers but relative to *N*_0_ of the most concentrated sample in the dilution series. **σ^2^**: Random variation around regression lines. ***F_Cq_***: Fluorescence at threshold in arbitrary LightCycler units (×100). ***k*** is the proportionality factor between *N_Cq_* and *F_Cq_* (*N_Cq_*/*F_Cq_*) and ***K_pr BP_*** the amplicon-length-corrected value. Parameters are comparable across days and targets since the relation between absolute and relative copy numbers is preserved.

### Run-Internal Mini-Standard curves (RIMS) simulation

To examine the usability of RIMS and RIMS-based drqPCR, we simulated a large number of individual quantifications from actual real-time PCR data. First, we combined the raw data (*C_q_* and *N*_0_) of the six 28-sample standard curves were used to generate 8,694 different RIMS. Each RIMS was based on raw data from two different concentrations of the same 28-sample standard curve data. In other words, each concentration was perceived as a separate RIMS sample (see results). The relative target concentration of a concentration-pair was denoted *C*. RIMS-concentration-pairs could be chosen in 7·(7−1)/2 = 21 different ways from each 28-sample standard curve data set and in 6·21 = 126 ways using all six data sets. For each of the seven concentrations of a 28-sample-SC, four *C_q_*-replicates were available. To simplify our simulation, we decided to use an equal number of *C_q_*-replicates for the two concentrations in the individual RIMS. Thus, for a given RIMS-concentration-pair, 4!/(1!·(4−1)!))^2^+4!/(2!·(4−2)!))^2^+4!/(3!·(4−3)!))^2^+4!/(4!·(4−4)!))^2^ = 69 different RIMS could be created, for a grand total of 8,694 ( = 126·69) RIMS using all six 28-sample-SC data sets. For each RIMS, the *α* (intercept) and *β* (slope) were estimated by conventional linear regression with *C_q_*s as the predictor variables and the relative concentrations as outcome variables.

### RIMS-based drqPCR simulation

The constructed RIMS were used in simulations of 2,500,848 direct relative quantifications (i.e. quantifications without reference to a control sample). To simplify, unicate quantifications were applied (only one *C_q_*-measurement of each target per quantification). The *C_q_*-measurements included in the applied RIMS were not quantified using the particular RIMS. Also, a RIMS-set, i.e. one of each target, is necessary for quantification. Therefore, (28−*m*)·(28−*n*) different unicate quantifications cound be constructed from a RIMS-set (*m* and *n* being the number of *C_q_*-measurements in the two RIMS). Because more than 18 million RIMS-sets could be constructed in this manner ( = (8,694/2)^2^), we introduced the constraint that each RIMS-set of ERV1 and TURPLE1 must encompass *C_q_*-measurements of samples positioned similarly in the LightCycler carrousel (that is, if sample numbers 1, 2, 5, and 6 were used for the ERV1 RIMS, then the same sample numbers were used for the TURPLE1 RIMS. In this way, 4,347 ( = 8,694/2) RIMS-sets were generated. The *α* and *β* of these sets were used with **Eq. R.1** and **Eq. R.2** for direct relative quantification of ERV1 and TUPLE1 in the remaining samples in the run that was not included in the RIMS. The total number of quantifications were 2,500,848 ( = (7·(7−1)/2)·(4^2^·(28−1·2)^2^+(4·3/2)^2^·(28−2·2)^2^+4^2^·(28−3·2)^2^+1^2^·(28−4·2)^2^)·3). To facilitate evaluation of accuracy and precision, we normalized the individual ratio-estimate by the theoretical ratio. The theoretical ratio was inferred from the quantified samples relative concentration based on data from the construction of the dilution series. This parameter, *R_is,Norm_*, ideally equals 1. Any systematic discrepancy reflects inaccuracy whereas stochastic variation around the mean estimate reflects precision.

For the evaluation of double-ratio drqPCR, a combined measure of concentration difference between interest- and virtual control sample in the individual quantification was calculated:
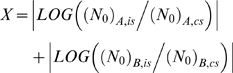
(M.3)


### 2*^ΔΔCq^*-based quantifications

Double-ratio-based quantification was used (**Eq. I.4**, *E_A_* = *E_B_* = 1). Samples of identical position of ERV1 and TUPLE1 for each serial dilution were used as control samples, and the remaining combinations of samples used as interest samples. This provided 61,236 (27^2^·28·3) double-ratios to be estimated. These were normalized by the theoretical ratio as above. *X*-values were determined as above.

### External-SC-based quantifications

The external-SC-based quantifications were done as the 2*^ΔΔCq^*-based quantifications, but with efficiency corrections based on external SCs of **Table 1**. Thus, 244,944 (27^2^·28·3·4) normalized double-ratios were calculated.

### Statistical analysis

Regression analysis was based on least-squares methods and *t*-distributions. Probability testing of variance similarity (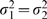) was based on the *F*-distribution: *F_obs_* = largest variance estimate/smallest variance estimate, degrees of freedom being (*f*_1_,*f*_2_) and two-tailed *p*-values = 2·*P*(*F*≥*F_obs_*). Significance level was set to 0.001 to avoid importance of mass significance.

## Results

### Direct relative quantitative PCR (drqPCR)

To estimate *R_is_* directly (without reference to a control sample), the unknown (*N_Cq_*)*_A_*/(*N_Cq_*)*_B_*-term presents a challenging. A simple solution is to perform real-time PCR on a sample containing *A* and *B* in equal concentrations. The (*N_Cq_*)*_A_*/(*N_Cq_*)*_B_*-term can then be estimated from the *C_q_*-data by rearrangement of **Eq. I.2**, provided that *E*-estimates are available (e.g. from standard curves). More elegantly, (*N_Cq_*)*_A_* and (*N_Cq_*)*_B_* can be inferred from the intercept of *A*s and *B*s standard curves (*α* = LOG (*N_Cq_*), cf. **Eq. M.1**). If the underlying relation between relative and absolute scales is similar for *A* and *B*, then a meaningful estimate of the (*N_Cq_*)*_A_*/(*N_Cq_*)*_B_*-term can be determined from the intercepts. Similar scales can be attained simply by constructing the standard curves from a common sample containing same concentrations of *A* and *B*. If such standard curves is used then it is unnecessary to estimate the actual (*N_Cq_*)*_A_*/(*N_Cq_*)*_B_*-term. Instead *R_is_* can be estimated as a difference of regression estimates (from **Eq. M.2**):
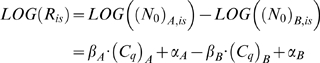
(R.1)The benefit of using regression estimates is that simple statistics can be applied to determining errors of LOG (*R_is_*) (see **Appendix S1** section 2). A sample containing *A* and *B* in equal concentrations is sometimes available. If not, the sample can be constructed; for example, by cloning *A* and *B* into the same plasmid. Instead of this somewhat cumbersome cloning approach, we recommend joining the targeted PCR products in a fusion-PCR product containing *A* and *B* in equal stoichiometry. This fusion-PCR product can be expanded endlessly by PCR and allows standard curves of maximal dilution ranges. A fusion-PCR product of ERV1 and TUPLE1 was constructed as a proof of principle and used in the present study (see the **Materials and Methods**-section).

### Run-Internal Mini Standards (RIMS)

**Figure 1:**
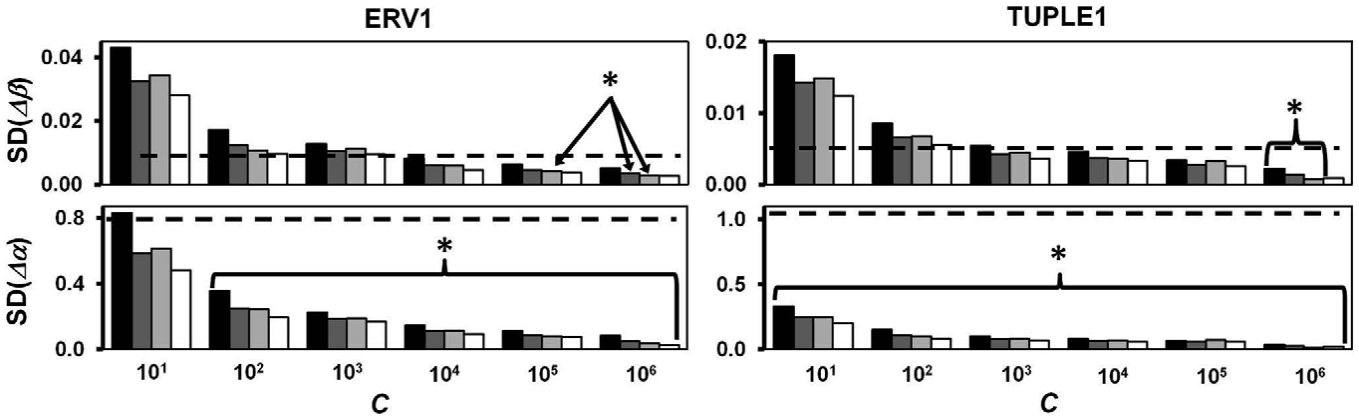
Precision of RIMS-based α- and β- estimates. RIMS-based parameters were referenced by subtraction to the corresponding estimates of the full internal standard curves. Standard deviation (SD) of *Δα*s and *Δβ*s were used to describe the precision of RIMS. Data were split according to target, *C*, and number of RIMS-sample replicates (1: black, 2: dark grey, 3: light grey or 4: white). The total number of RIMS estimates for each target can be determined from the specific *C* value and the number of RIMS replicates (*m*) as follows: *n* = (7−LOG (*C*))·(4!/(*m*!·(4−*m*)!))^2^·3. The precision of parameters of external standard curves (*n* = 6 in each figure) was calculated in a similar manner and is shown as broken, black horizontal lines. An asterisk indicates where RIMS demonstrated significantly better precision than external standard curves (*p*<0.001).

The accuracy of **Eq. R.1** hinges on use of valid estimates of slopes and intercepts. These parameters can vary significantly between targets but also between PCR runs of the same target (**Tabel I**). Therefore, target-specific and run-specific parameters are preferable. We hypothesized that internal standard curves based on fewer samples are preferable over larger, external standard curves. This hypothesis was confronted as follows:

Initially, we sought an optimal sample composition strategy for RIMSs. The composition should minimize the errors of regression estimates of LOG ((*N_0_*)*_A,is_*) and LOG ((*N_0_*)*_B,is_*) in **Eq. R.1**. Hellemans et al point out the error of the slope in linear regression is reduced by expanding the range of the dilution and including more measurements points [13]. A similar principle applies to the error of regression estimates (cf **Eq. S2.1** in **Appendix S1**). A large number of measurement points are not desirable with RIMS. However, it is deductable that predictor variable extremes reduce the error more effectively than those close to the predictor variable mean. We therefore based our strategy solely on “extreme concentrations” and used only two samples, of relative concentration *C*, for our RIMSs. Our next step was to determine the importance of *C*-size and number of replicate analyses of the two RIMS-samples for the precision of *α*- and *β*-estimates. The total of 8,694 different RIMS was constructed from the raw data of six 28-sample standard curve data sets presented in **Table 1** (see **Material and Methods**). The precisions of the RIMS-based *α*- and *β*-estimates, compared with those of external standard curves, are presented in **Figure 1**. In particular, we found that precision was increased by increasing values of *C*. The benefit of increasing the RIMS-sample replicate number was less obvious.

### RIMS-based single ratio drqPCR

**Figure 2:**
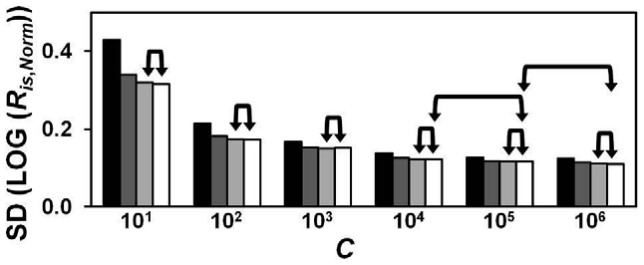
Precision of RIMS-based single ratio drqPCR. The importance of *C*, RIMS sample replicate number (1: black, 2: dark grey, 3: light grey, or 4: white) for quantitative precision (SD (LOG (*R_is_*_,*Norm*_))). The 8,694 sets of RIMS-derived parameters (*α* and *β*) of similar *C* and RIMS sample replicate number were paired for ERV1 and TUPLE1. Each of the 4,347 paired RIMS-parameter sets were used to calculate all possible run-internal (unicate) LOG (*R_is_*) from the remaining individual *C_q_*s not included in the specific RIMS pair. Each LOG (*R_is_*) was normalized by subtracting the logarithmic transformed actual target ratio (determined from the sample's position in the serial dilutions). This provided a total of 2,500,848 LOG (*R_is_*_,_Norm)s. These were sub grouped according to *C* and RIMS replicate number. The SD of subgroups is illustrated. The number of *R_is_*_,*Norm*_s in each subgroup is calculable as: (7−LOG (*C*))·(4!/(*m*!·(4−*m*)!))^2^·3·(28−*m*)^2^. A ten-fold increase of *C* as well as introduction of an additional RIMS sample replicate provided significantly (p<0.001) better precision with the exceptions indicated by arrows in the figure.

Next, we investigated the quantitative precision and accuracy of our two approaches when used in combination. In total, 2,500,848 unicate quantifications were determined from the raw data of the six 28-sample data sets. The quantitative precision is summarized in **Figure 2**. Increases in *C* and RIMS-sample replicate numbers both generally conferred significant precision improvements. However, the effect of using four as opposed to three RIMS-sample replicates was insignificant. The accuracy was unaffected by *C* or RIMS-sample replicate number and ranged between 94% and 110% of the true target ratios. **Double-ratio drqPCR** as argued in the introduction, the double-ratio approach (**Eq. I.4**) can provide *R_is_*-estimates **if** the control sample contains *A* and *B* in equal concentrations. Using **Eq. R.1**, LOG (*R_is_*) can be estimated as:
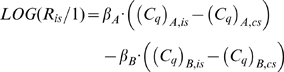
(R.2)A prerequisite is that *α*s and *β*s are constant between interest and control samples for each target.

Some heterogeneity is evident in a comparison of single-ratio to double-ratio based drqPCR (**Eq. R.1** and **Eq. R.2**). Fewer different samples are required in the former approach. This confers fewer sources of errors to *R_is_*. However, erroneous *β* are expectedly more critical in the single-ratio approach, because *β* is multiplied by larger values (full *C_q_*s as opposed to Δ*C_q_*s in the double-ratio approach). In the double-ratio approach, errors in *β*-estimates become less critical when Δ*C_q_*s decreases. A potential drawback of double-ratio drqPCR is that more capacity is required. However, this may be circumvented by using one of the RIMS samples as control sample also. The control sample should be chosen as the RIMS sample providing the smallest Δ*C_q_*s for the individual target in the given quantification. Naturally, one RIMS sample may be closest to *A* while another is closest to *B*. Acknowledging, a “virtual control sample” can be constructed for the specific interest sample-to-be-quantified comprised of *C_q_*- and *N*_0_-data from the closest RIMS sample (chosen target-specifically). Correction for the situation where (*N_0_*)*_A,cs_*≠(*N_0_*)*_B,cs_* is remedied using by the following equation:
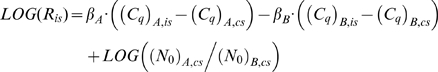
(R.3)


### Single- vs. double-ratio drqPCR

**Figure 3:**
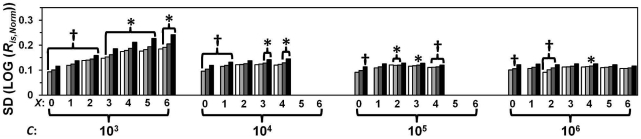
Comparison of single- vs. double-ratio drqPCR. Bars illustrating the precision of RIMS-based and double-ratio-based drqPCR as a result of RIMS sample replicate number (1: black, 2: dark grey, 3: light grey, or 4: white), *C*, and *X* (**Eq. M.3**). The precision of drqPCR based on double-ratios was compared with the precision of single-ratio-based drqPCR (**Figure 2**). Asterisk and crosses indicates significantly (*p*<0.001) better precision in quantification based on single-ratios and double-ratios, respectively.

We compared the precision and accuracy of quantification by single- and double-ratio drqPCR in the 1,190,880 possible quantifications from the six 28-samplestandard curve data sets (*C*≥10^3^). We expected that increasing the Δ*C_q_* of interest sample and virtual control sample would decrease the precision of double-ratio drqPCR. Data were therefore split according to *X*, which is a measure of this distance (**Eq. M.3**). Results are presented in **Figure 3**. For low *C*s (10^3^–10^4^), the double-ratio approach was more precise when target concentrations of control and interest samples were close (X≤2, see **Figure 3**). Single-ratio-based drqPCR was more precise for larger concentration differences. For large *C*s (10^5^ to 10^6^), we observed no remarkable differences between the approaches. The accuracy of both approaches was within ±8%.

### Comparison of RIMS-based drqPCR, the 2*^ΔΔCq^*-approach, and relative quantification based on external standard curves

**Figure 4:**
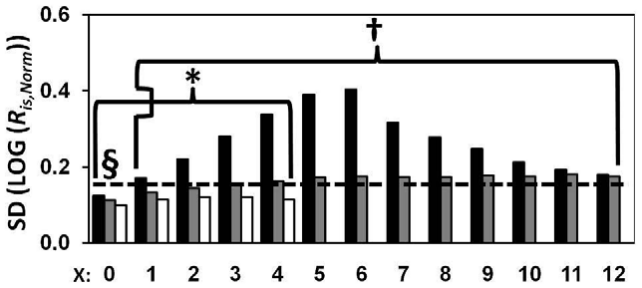
Comparison of the quantitative precision of drqPCR based on RIMS, external standard curves, or the 2^ΔΔCq^-approach. Illustration of the precision of drqPCR (SD (LOG (*R_is_*_,*Norm*_))) based on the 2*^ΔΔCq^*-approach (black bars), external standard curves (dark grey bars), and RIMS (exemplified by *C* = 10^5^ and RIMS samples analyzed in duplicates). RIMS parameters were used in single-ratio drqPCR (horizontal broken, black line) and double-ratio drqPCR (white bars). External standard curve-based and 2*^ΔΔCq^*-based drqPCR where by the double-ratio approach only. As control sample data, we used the run-internal, identical-sample-position *C_q_*s of ERV1 and TUPLE1. The remaining run-internal combinations of ERV1 and TUPLE1 for a given control sample pair were treated as interest samples. The number of quantifications was 61,236 (27·28·3) for the 2*^ΔΔCq^*-approach and 244,944 (27^2^·28·3·4) for use of external standard curves. The LOG (*R_is_*)s of the conventional approaches were normalized as those determined by RIMS-based drqPCR (**Figure 2**, legend). Asterisk and crosses indicates significantly (*p*<0.001) better precision in quantification based on single-ratios and double-ratios compared to conventional approaches, respectively. The § at X = 0 indicates the only *X*-value where external standard curves offered significantly better precision than single-ratio-based drqPCR.

The data of the six 28-samplestandard curves were used to generate 61,236 and 244,944 different quantifications by the 2*^ΔΔCq^*-approach and based on external standard curves, respectively. Quantifications were based on **Eq. I.4**. Control samples containing ERV1 and TUPLE1 in equal concentrations were used. Results were normalized by the theoretical ratio and compared with those obtained from RIMS based single- and double-ratio (**Figure 4**). RIMS based drqPCR offered potential for significantly better precision, regardless of *X*-size. The accuracy of the conventional approaches was overall within ±3% but ±7% if data were split according to the used standard curves.

## Discussion

Real-time relative quantitative PCR in clinical settings is hampered by a lack of accurate and precise approaches to estimate the ratio between nucleic acid sequences without reference to a control sample. Also, conventional approaches for estimating internal PCR parameters are problematic in low capacity PCR machines. This paper concerns the establishment and examination of two new approaches for real-time quantitative PCR; namely RIMS and drqPCR. RIMS concerns estimation of run-internal specific PCR parameters, such as efficiency, from a minimum of samples. The drqPCR is a universal strategy for estimation of ratios directly in the sample, alleviating the need for control samples and therefore ideal for analysis of unpaired samples. We compared RIMS and drqPCR with conventional methods on a common data set. This data set was generated from samples with known target ratios. Therefore, both the precision and accuracy of the approaches could be evaluated.

Separately, RIMS gives target-specific and run-specific estimates of the standard curve's slope and intercept (measures of *N_Cq_* and *E*, respectively). Estimates determined for the specific run are obviously preferable to those determined in separate runs. Run-specific differences of *N_Cq_* for the targets can be corrected by inclusion of calibrator samples. However, run-to-run differences in *E* are not corrected by use of calibrator samples and are critical to the use of external standard curves. It is evident from our data that run-to-run differences of *E* are to be anticipated (**Table 1**). Testing the *β*s of the same target for significant differences over days discloses dissimilarity in 3 of the possible 6 comparisons (*p*<0.001 for ERV1 on day 1 vs. 3, TUPLE1 on day 1 vs. 3, and 2 vs. 3).

From Real-Time PCR data, we produced 8,694 individual RIMS. Not surprisingly, the number of RIMS-sample replicates and the value of *C* were of immense importance for precision (**Figure 1**). Compared with standard curves, RIMS provided the potential for attaining estimates of the highest precision. Obviously, a potential explanation could be large run-run external standard curve-variation in our study. Rutledge and Cote [10] used a model comparable to ours, in which a PCR product was serially diluted and subjected to real-time PCR with two different primer sets five times. They reported *E* CVs of 2.2% and 2.1% for five repeated standard curves for each of two targets. In comparison, *E* CVs of our study were 2.9% (ERV1) and 2.5% (TUPLE1). The CVs of *N_Cq_* in the study of Rutledge and Cote were 19.0% and 14.7%. *N_Cq_*-detection was based on constant fluorescence threshold. We based our threshold setting on an error-minimizing strategy for the individual standard curve. Correction of our *N_Cq_* -data by fluorescence intensity at *C_q_* enables comparison. Threshold-corrected *N_Cq_* CVs of our study were 12.4% and 26.3% (data from **Table 1**). However, the CVs of Rutledge et al do not include the variation associated with the construction of the dilution series, insofar as all standard curves were generated from sequential analysis of only one dilution series. In this light, our raw data are at least comparable in quality.

In our examination of the precision of RIMS-based estimates, the estimates were compared to the corresponding estimate of the internal standard curves. We found a close approximation (that is, a high precision) of the large-*C*-based RIMS estimates to the 28-sample internal standard curves' (**Figure 1**). We perceive this as indicative of RIMS potential for high accuracy. Furthermore, the result illustrates the redundancy of the intervening data points. RIMS may therefore also be considered as a cost-saving alternative in high-capacity machinery.

The following points should be considered when constructing the RIMS in practice. First, RIMS samples should be selected from the dilution series based on a preliminary standard curve to maximize *C* (≥10^5^) while preserving linearity. Second, very dilute samples should be avoided completely (**Appendix S1**, section 1). Third, the chosen samples should be aliquoted and stored. Fourth, two or more replicate *C_q_*-estimations of each RIMS sample are preferable. Fifth, the type of template (e.g. PCR-product, cDNA, or genomic DNA) chosen for RIMS-samples should permit appropriately sized *C*s. Direct estimation of nucleic acid sequences ratio is attractive in many settings, yet difficult to obtain. The problem, associated with the (*N_Cq_*)*_A_*/(*N_Cq_*)*_B_*-term (cf. **Eq. I.2**), can be dealt with in several ways. The simplest is to ignore it altogether. This is the case if ordinary relative standard curves are used. Such an approach confers systematic errors of magnitudes defined by the inverse of the (*N_Cq_*)*_A_*/(*N_Cq_*)*_B_*-term. In our example, we would have attained between 1.1-fold and 2.6-fold errors for same-day quantifications and as much as 20-fold errors for quantifications across days (cf. *N_Cq_*-values in **Table 1**). Another approach is to rely on experimental conditions assumed to ensure equality of (*N_Cq_*)*_A_* and (*N_Cq_*)*_B_*. If equality is indeed ensured, the (*N_Cq_*)*_A_*/(*N_Cq_*)*_B_*-term can be safely ignored. A frequent perception in Real-Time PCR literature is that a similar detection threshold for different targets automatically ensures similar copy numbers at detection [8], [10], [14], [15]. As described earlier, this is unreliable because the proportionality factor between copy numbers and fluorescence intensity (*k*) can differ between targets. The magnitude of the systematic error associated with the approach can be assessed as the inverse of the targets *k*-ratio. With this approach, our results would have been off by factor of 1.2 to 1.6 or 2.0 to 2.7 with or without target length corrections, respectively (cf. *k*-values of **Table 1**). Although we observed a great deal of variability of the *N_Cq_*s across both days and targets, this does not prove the existence of a universal phenomenon. However, our findings do illustrate the importance of expecting variation. This definitely also applies if a common threshold setting is defined. Quantitative-methods with insufficient corrections of (*N_Cq_*)*_A_*/(*N_Cq_*)*_B_*-term variability can provide results of reasonable precision. But the systematic error is never spotted unless samples of known ratio are quantified. A third and more qualified approach is to use classical absolute quantifications [16]. A drawback of absolute quantification is the considerable effort required to generate samples of known absolute target concentration. Typically, the targets are cloned into plasmids, which are subsequently biologically expanded and purified. The ensuing measurements of DNA concentrations and the derivation of absolute target concentrations are both prone to errors.

Instead, we advocate approaches based on internal, relative standard curves (derived from samples containing the targets of interest in equal amounts) and use of regression estimates. This ensures both target-specific and run-specific corrections of the underlying variable (*N_Cq_*)*_A_*/(*N_Cq_*)*_B_*. Ligated PCR products of the targets are the ideal theoretical choice. This generally applicable method has the potential for attaining the largest *C*s and absolutely defining the targets' stoichiometry. For data handling, we suggest two different approaches: single-ratio or double-ratio-based drqPCR (**Eq. R.1** and **Eq. R.3**, respectively). In the double-ratio-based approach, a virtual control sample is constructed from the RIMS samples already used for *β*-estimations. Such “recycling” of RIMS-samples is not problematic, inasmuch as the samples are used to estimate two independent quantities. The known relative concentration between the RIMS-samples (e.g. *C_A_* = (*N*_0_)***_A_***_*,RIMS1*_/(*N*_0_)***_A_***_*,RIMS2*_) is used for *β*-estimations, whereas the relative concentration of the targets of the virtual control sample (e.g. (*N*_0_)***_A_***_*,RIMS1*_/(*N*_0_)***_B_***_*,RIMS1*_)) is used in corrections of the (*N_Cq_*)*_A_*/(*N_Cq_*)*_B_*-term. Steps to reduce the error of *R_is_*-estimation are of general importance. This is obvious in clinical settings but also applies to experimental comparison of groups. The larger the variance of a group mean, the more individuals, cell cultures or such, must be included to demonstrate a given significant difference between groups. With this in mind we allocated some efforts to choose the ideal raw data sampling approaches. *C_q_*-sampling by the FP-approach offered significantly better precision than the second derivatives maximum-approach, whereas estimations of relative target concentrations in standard curves based on weight as opposed to volumes was negligible (and insignificantly) better (see **Appendix S1** section 3 and **Figure S2**, **Figure S3**, and **Figure S4**).

We evaluated drqPCR based on RIMS in quantifications of samples containing the quantified targets in known stoichiometry. This model permitted us to evaluate both quantitative precision and accuracy. Not surprisingly, we found that increasing *C* and the number of RIMS replicates increased the precision significantly (**Figure 2** **–** **3**). However, *C* was the most important parameter for improving precision. More than two replicates of each RIMS-sample conferred only minimal improvements of precision. Accuracy was within ±8%. Finally, we compared precision and accuracy of drqPCR based on the 2*^ΔΔCq^*-approach, external standard curves, and RIMS. RIMS-based drqPCR demonstrated the largest potential for precision (**Figure 4**). The accuracies of the conventional approaches were comparable. The conventional approaches were conducted in a double-ratio manner where *R_is_*s were normalised by the ratio derived from analysis of a sample containing the targets in equal concentrations, to maximize accuracy. When using our two approaches in practice, we suggest inclusion of an intermediate RIMS sample. The purpose is dual: to permit evaluation of linearity within the specific RIMS, and to provide more samples available for “virtual controls”. Also, the *C_q_* of samples to be quantified should be estimated in duplicates or more. For practical data handling, we have included an Excel-based spreadsheet in the supplementary material (**Algorithm S1**).

Use of drqPCR has other beneficial side-effects. The principle renders calibrator samples (or reference-control samples) superfluous. Calibrators are a necessity when interest and control samples are not in the same PCR-run [3]. Their purpose is to correct for run-to-run differences of targets *N_q_*-value. Briefly, the calibrator sample contains the targets-to-be-quantified and is PCR-expanded both in runs of controls and interest samples. Subsequently, interest and control data are made comparable by dividing each with the calibrator data of their respective runs. In drqPCR, data of control and interest samples are always immediately comparable, provided that the same RIMS samples are used in runs. Avoidance of calibrators is attractive to minimize the sources of errors of *R_is_*. The importance of drqPCR can be stretched further. Another important effect is that results obtained by drqPCR are immediately comparable between different labs. Thus, the problem of lab-to-lab comparability is avoided completely.

In summary, we suggest that RIMS and drqPCR be used separately or combined for relative quantifications of high precision and accuracyThe drqPCR allows determination of *R_is_* directly in the sample, and RIMS can replace external standard curves.

## Supporting Information

Appendix S1 (0.12 MB DOC)

Figure S1 The association of replicate-*C_q_* spread and *N_0_C_q_* residuals were calculated (difference of individual *C_q_* and mean *C_q_* for the target, day and target concentration) and plotted against the sample number (1–8) of the dilution series. Levene's test for equality of *C_q_*-residuals' variances between groups where conducted. Inclusion of data of the eighth samples was associated with heteroscedasticity (*p*: 6·10^−11^) whereas exclusion conferred homoscedasticity (*p*: 0.66). NB: On this single occasion, we made two modifications to our *C_q_*-sampling approach. Firstly, noise bands were set manually for 3 out of 6 data sets. This was necessary to ensure that thresholds were not set in the lower non-logarithmic phases. Secondly, *C_q_*s of all eight concentrations of the dilution series were used in the minimizing error strategy.
(0.21 MB TIF)

Figure S2 Precision of RIMS based single-ratio drqPCR with *C_q_*-sampling by SDM and FP (black and white bars, respectively) for different values of *C* (RIMS samples analysed in duplicates). *C_q_*-sampling by FP prompted significantly (*p*<0.001) better quantitative precision for all values of *C*.
(0.11 MB TIF)

Figure S3 Precision of double-ratio-based qPCR by the 2*^ΔΔCq^*-approach (black and dark grey bars) or from external standard curves (light grey and white bars) for variable sizes of *X* (defined in main text). *C_q_*-sampling was by SDM (black and light grey bars) or FP (dark grey and white bars). Quantifications based on *C_q_*-sampling by FP were more precise for both approaches of efficiency estimations for all *X*-values. The improvements were significant (*p*<0.001) except for external standard curves and *X* of 5 (*p*: 0.035) and 6 (*p*: 0.066) (indicated by asterisks).
(0.18 MB TIF)

Figure S4 Quantitative precision of single-ratio drqPCR based on RIMS (duplicate analysis) with estimations of *C* based on pipetted weights (white and dark grey bars) or volumes (black and light grey). Weight as opposed to volume based *C*-estimation provided minute improvements of precision for all *C*s regardless of *C_q_*-sampling approach (SDM: black and dark grey, FP: light grey and white). However, improvements were insignificant except where indicated by an asterisk (*p*: 0.0002). Analysis of RIMS samples in replicates of 1, 3, and 4 demonstrated similar findings.
(0.13 MB TIF)

Algorithm S1 Algorithm for practical use of RIMS based drqPCR.
(1.26 MB XLS)
