# Supplementary material for: Real-Time Relative qPCR without Reference to Control Samples and Estimation of Run-Specific PCR Parameters from Run-Internal Mini-Standard Curves
Source: PLoS One. 2010 Jul 22;5(7):e11723. doi: 10.1371/journal.pone.0011723 (PMC2908630; doi:10.1371/journal.pone.0011723)
Supplement: Appendix S1 — (0.12 MB DOC) [file pone.0011723.s001.doc]

1. **Avoidance of heteroscedasticity by exclusion of very dilute samples**

Application of standard curves relies on simple linear regression analyses. In such analyses the random error about the regression line must demonstrate equal variance (homoscedasticity). We hypothesised that use of very dilute samples in standard curves are critical to this prerequisite. The reason is that stochastic pipetting errors expectedly will be of far larger relative importance for very low concentrations.

We examined the practical importance of target concentration for *C_q_­*-spread of replicates. The *C_q_*-variance of the most dilute sample was by far the largest (**Figure S1**). The *C_q_*-residuals for both targets and all days were grouped according to target concentration (*N*_0_). Group variances were examined for equality using Levene´s test with or without data of the most dilute concentration (sample number 8 of dilution series, *N*_0_=10^-7^). Inclusion of the data group obtained from the eighth sample of the dilution series introduced heteroscedasticity between data groups (*p*= 6*10^-11^) whereas exclusion entailed homoscedasticity amongst the remaining (*p*= 0.66). Based on this finding, we omitted data of the lowest concentration from the all other parts of this study.

Based on the above reasoning and our empirical data, we emphasise that inclusion of very dilute samples in standard curves generally must be avoided. Data quantity of an individual standard curve is not likely to justify application of tests of heteroscedasticity (e.g. Bartlett´s -, Levene´s -, or Brown-Forsythe tests). So in practical experiments, a more pragmatic approach might be preferred. Based on intuition and the above findings we suggest the following: Data are only to be included in the standard curve if a less concentrated sample (at least 10-fold) provided *C_q_*s for all replicates. For practical purposes, this ensures that critically dilute samples are not included. The identification of qualified samples for RIMS can be conducted in a preliminary standard curve.

1. **Error of drqPCR derived LOG (*R_is_*)-estimates**

As detailed in the text, *R_is_* can be estimated by **Eq. R.1** based on LOG (*N*_0_)-regression estimates the targets to be quantified. Obviously, error is associated with each LOG (*N*_0_)-regression estimate due to the errors of *α*- and *β*. Formulas for estimating such standard errors are available in statistical textbooks (Bland M [1] modified for this papers terminology):

**Eq.S2.1**: $SE_{regr}\left(LOG\left(N_{0}\right)\right)=\sqrt{s^{2}\left(\frac{1}{n}+\frac{\left(C_{q}-\overline{C_{q},_{SC}}\right)^{2}}{\sum\left(\left(C_{q},_{SC}\right)_{i}-\overline{C_{q},_{SC}}\right)^{2}}\right)}$

Where *n* is the number of samples in the standard curve (RIMS) and $s^{2}$ is estimated as:

**Eq. S2.2**: $s^{2}=\frac{1}{n-2}\left(\sum\left(LOG\left(N_{0},_{SC}\right)_{i}-\overline{LOG\left(N_{0},_{SC}\right)}\right)^{2}-\beta^{2}\sum\left(\left(C_{q},_{SC}\right)_{i}-\overline{C_{q},_{SC}})^{2}\right)\right)$

The SE of *R_is_*, associated with the regression estimates, can be estimated by combining the SEs of the two subtrahends:

**Eq.S2.3**: $SE_{regr}\left(LOG\left(R_{is}\right)\right)=\sqrt{SE_{regr}\left(LOG\left(N_{0}\right)_{A}\right)^{2}+SE_{regr}\left(LOG\left(N_{0}\right)_{B}\right)^{2}}$

The SE estimated by **Eq.S2.1** does not take the error of the particular *C_q_*-estimation of the sample-to-be-quantified into account. In many applications of regression estimates, the error associated with the actual estimation of the predictor variable is of neglectable importance compared to the error associated with applying the regression equation. However in Real-Time PCR, even subtle variation in *C_q_*-replicates can be important (since *C_q_*s are exponents in exponential functions). In line with this, *C_q_*s of samples-to-be-quantified are typically estimated in dupli- or triplicates out of the rationale that increasing *C_q_*-replicate number reduces mean-*C_q_* error and ultimately mean-LOG (*N*_0_) error. The SE of the mean LOG (*N*_0_)-estimate can be determined from the standard deviation (*SD*) of individual LOG (*N*_0_)-estimates and their number:

**Eq.S2.4**: $SE_{repl}\left(LOG\left(N_{0}\right)\right)=\frac{SD}{\sqrt{n}}$

Individual LOG (*N*_0_)-estimates are estimated from their underlying *C_q_*s calculated with **Eq. M.2**.

For LOG (*R_is_*)s estimated from **Eq. R.1 w**e propose to approximate combined SE taking both errors associated with regression (**Eq.S2.1**) and *C_q_*-errors of the-sample-to-be-quantified (**Eq.S2.4**) into account:

**Eq.S2.5**: $SE_{regr}\left(LOG\left(R_{is}\right)\right)=$

$$\sqrt{SE_{regr}\left(LOG\left(N_{0}\right)_{A}\right)^{2}+SE_{regr}\left(LOG\left(N_{0}\right)_{B}\right)^{2}+SE_{repl}\left(LOG\left(N_{0}\right)_{A}\right)^{2}+SE_{repl}\left(LOG\left(N_{0}\right)_{B}\right)^{2}}$$

LOG (*R_is_*) can also be estimated from **Eq. R.3**. As discussed in the text, the difference to estimation by **Eq. R.1** is in that the intercepts (and thus the errors associated with them) have cancelled out. On the other hand, the samples that are to have estimated *C_q_*s have doubled. We propose to approximate SE of LOG (*R_is_*) in this setting by a strategy similar to the above. The combined SE is perceived comprised of errors associated with using regression estimates and the errors of *C_q_*-estimations. Thus the SE can be determined from **Eq.S2.5** with two modifications 1) two additional terms are in the radicand: $SE_{repl}\left(LOG\left(N_{0}\right)_{A},_{cs}\right)^{2}$ and $SE_{repl}\left(LOG\left(N_{0}\right)_{B},_{cs}\right)^{2}$and 2) only the error of *β* (and not that associated with *α*) is included in SE_regr_s:

**Eq.S2.6**: $SE_{regr}\left(LOG\left(N_{0}\right)\right)=SE\left(\beta\right)\cdot\left|\left(C_{q}\right)_{is}-\left(C_{q}\right)_{cs}\right|$

The error estimate of *β* can be estimated from:

**Eg.S2.7:** $SE\left(\beta\right)=\sqrt{\left(\frac{s^{2}}{\sum\left(\left(C_{q},_{SC}\right)_{i}-\overline{C_{q},_{SC}}\right)^{2}}\right)}$ , $s^{2}$ is estimated from **Eq. S2.2**

1. **Importance of raw data sampling approaches**

Raw data for construction of standard curves (*C_q_*s and target concentrations) and for samples to be quantified (*C_q_*s) can be sampled by various approaches. Naturally, the accuracy and precision of the sampling approaches are ultimately reflected in the quantitative accuracy and precision.

***C_q_*-sampling** *C_q_* is typically sampled as the interpolated (arbitrary) PCR cycle where a defined threshold of fluorescence intensity is acquired. The *C_q_*-data sampling in this study (described in the **Material and Methods**-section of the main text) is such an approach (abbreviated FP). Other approaches are used. Commonly, users of the LightCycler favours the second-derivatives-maximum (SDM) approach. In the SDM approach, software automatically estimates the *C_q_* from the kinetics of the individual analysis´ fluorescence over cycles. Practically, the point of maximal fluorescence-intensity acceleration is determined and the corresponding arbitrary cycle estimated. SDM is user friendly, but errors introduced by the additional algorithm might be anticipated. We initially evaluated the errors of our 28-sample standard curves to select the mode of *C_q_*-sampling. Data based on SDM is presented in **Table SI**. SDM consistently provided larger errors than FP (cf main text **Table I**).

We examined the importance of *C_q_*-sampling approach quantitative accuracy and precision. *C_q_*s were determined by SDM and this data used to recalculate the 2,500,848 *R****_is_****_Norm_* described in **Results** of main text: **RIMS-based single ratio drqPCR**. A comparison of the two *C_q_*-sampling approaches quantitative precision is presented in **Figure S2**. For simplicity, only data obtained from RIMS based on two replicates of the RIMS-samples are presented (similar findings were made with 1, 3, and 4 RIMS sample replicates). Use of SDM was in our setting associated with significant (and important) loss of precision. Accuracy was unaffected. In a similar manor, we compared precision and accuracy of *R****_is_***,*_Norm_* for quantification based on external standard curves and the 2^ΔΔC^*^q^*-approach (**Figure S3)**). Similar findings were made.

We suggest that *C_q_*-sampling by SDM is used cautiously. If precision is of great importance, use of SDM must be demonstrated to provide comparable or better precision and accuracy than manual threshold setting. Such evaluation could be done by comparing the errors of internal standard curves (e.g. RIMS) for the *C_q_*-sampling approaches in question.

**Sampling of *N*_0_s for standard curves** The relative-target-concentration-data for standard curves are generally estimated from the volume-setting of the pipettes used for construction of the dilution series. However, these pipettes are typically calibrated based on weight of sampled volumes. The errors related solely to the pipette can therefore be avoided by applying weight data instead of expected volumes in the standard curves. Weight data of the pipetted volumes in the three, eight concentration serial dilutions of the present study is presented in **Table S2**. Pipetting errors propagate in the dilution series. To minimize importance of stochastic errors we chose a strategy of conducting each dilution step in triplicates. Use of volume data entailed that the estimated relative concentration was as much as 10 % off the best bid based on weight data (cf. **Table S3**).

However, weighing the sampled volumes is more labour intensive. To examine if it is worth while, we investigated the impact on precision and accuracy of *R****_is_***,*_Norm_*. The 2,500,848 quantifications were recalculated with use of *N*_0_,*_Rel_*-estimations of standard curves based on volumes or weights (**Figure S4**). Weight based standard curves offered only insignificant minute improvements of precision. Accuracy was comparable.

We conclude that the practical benefits of using weight data is unlikely to be of importance.

**Table S1**

| **Target** | **Day** |  | ***σ*^2^** | ***β*** | ***α*** | ***E*** | ***N_C_****_q_* |
| --- | --- | --- | --- | --- | --- | --- | --- |
| **ERV1** | **1** |  | **.0098** | **-.272** | **2.64** | **.869**  [.846;.891] | **433**  [332;565] |
|  | **2** |  | **.0235** | **-.274** | **2.74** | **.880**  [.845;.916] | **552**  [363;838] |
|  | **3** |  | **.0068** | **-.271** | **2.72** | **.865**  [.847;.884] | **530**  [423;663] |
|  |  |  |  |  |  |  |  |
| **TUPLE1** | **1** |  | **.0031** | **-.289** | **2.23** | **.946**  [.932;.960] | **169**  [147;194] |
|  | **2** |  | **.0032** | **-.287** | **2.25** | **.936**  [.922;.950] | **177**  [154;204] |
|  | **3** |  | **.0022** | **-.283** | **2.21** | **.917**  [.905;.928] | **161**  [143;181] |

**Legend to Table S1** Data of the six 28-sample standard curves generated from Real-Time PCR data based on *C_q_*-data sampling by SDM.

**Table S2**

|  |  | **Weight for pipette volume set at:** | | | | | | |
| --- | --- | --- | --- | --- | --- | --- | --- | --- |
|  |  | **20 μl** | | |  | **180 μl** | | |
| **Day** |  | **Mean** [mg] | ***n*** | **s.d.** [mg] |  | **Mean** [mg] | ***n*** | **s.d.** [mg] |
| **1** |  | 20.3 | 21 | 0.11 |  | 179.3 | 21 | 0.65 |
| **2** |  | 20.1 | 21 | 0.11 |  | 179.4 | 21 | 0.31 |
| **3** |  | 20.0 | 21 | 0.08 |  | 181.3 | 21 | 0.33 |

**Legend to table S2** Weights of the pipetted volumes in the three dilution series utilised in this study. Each dilution step was conducted in triplicates. This was to minimize the importance of stochastic errors. Each dilution series was conducted in seven steps and therefore contained eight concentrations.

**Table S3**

| **Step** |  | **Fold-dilution of per step** | | | |  | **Accumulated fold-dilution** | | | |
| --- | --- | --- | --- | --- | --- | --- | --- | --- | --- | --- |
|  |  | **Volume** | **Weight (day)** | | |  | **Volume** | **Weight (day)** | | |
|  |  |  | **1** | **2** | **3** |  |  | **1** | **2** | **3** |
| **1** |  | 10 | 9.80 | 9.91 | 10.1 |  | 10^1^ | 0.98∙10^1^ | 0.99∙10^1^ | 1.01∙10^1^ |
| **2** |  | 10 | 9.88 | 9.90 | 10.1 |  | 10^2^ | 0.97∙10^2^ | 0.98∙10^2^ | 1.02∙10^2^ |
| **3** |  | 10 | 9.84 | 9.93 | 10.1 |  | 10^3^ | 0.95∙10^3^ | 0.97∙10^3^ | 1.03∙10^3^ |
| **4** |  | 10 | 9.82 | 9.93 | 10.1 |  | 10^4^ | 0.94∙10^4^ | 0.97∙10^4^ | 1.03∙10^4^ |
| **5** |  | 10 | 9.91 | 9.89 | 10.0 |  | 10^5^ | 0.93∙10^5^ | 0.96∙10^5^ | 1.03∙10^5^ |
| **6** |  | 10 | 9.84 | 9.94 | 10.0 |  | 10^6^ | 0.91∙10^6^ | 0.95∙10^6^ | 1.04∙10^6^ |
| **7** |  | 10 | 9.87 | 9.91 | 10.1 |  | 10^7^ | 0.90∙10^7^ | 0.94∙10^7^ | 1.05∙10^7^ |

**Legend for Table S3** Volume and weight data of the three dilutions series utilized in this study (left side). Accumulated fold-dilutions are calculated from the data (right side).

REFERENCES

[1] M. Bland, An introduction to medical statistics, Oxford University Press, Oxford, 2000
